# Potential urinary function benefits of initial robotic surgery for rectal cancer in the introductory phase

**DOI:** 10.1007/s11701-021-01216-5

**Published:** 2021-03-16

**Authors:** Hiroshi Oshio, Yukiko Oshima, Gen Yunome, Mitsuyasu Yano, Shinji Okazaki, Yuya Ashitomi, Hiroaki Musha, Yukinori Kamio, Fuyuhiko Motoi

**Affiliations:** 1 Department of First Surgery, Yamagata University Hospital, 2-2-2 Iidanishi, Yamagata-shi, Yamagata-ken, 990-9585 Japan; 2 Department of Surgery, Sendai Medical Center, 2-11-12 Miyagino, Miyagino-ku, Sendai, Miyagi-ken 983-8520 Japan

**Keywords:** Robotic surgery, Laparoscopic surgery, Rectal cancer, Propensity score analysis

## Abstract

We aimed to evaluate the advantages and disadvantages of initial robotic surgery for rectal cancer in the introduction phase. This study retrospectively evaluated patients who underwent initial robotic surgery (*n* = 36) vs. patients who underwent conventional laparoscopic surgery (*n* = 95) for rectal cancer. We compared the clinical and pathological characteristics of patients using a propensity score analysis and clarified short-term outcomes, urinary function, and sexual function at the time of robotic surgery introduction. The mean surgical duration was longer in the robot-assisted laparoscopy group compared with the conventional laparoscopy group (288.4 vs. 245.2 min, respectively; *p* = 0.051). With lateral pelvic lymph node dissection, no significant difference was observed in surgical duration (508.0 min for robot-assisted laparoscopy vs. 480.4 min for conventional laparoscopy; *p* = 0.595). The length of postoperative hospital stay was significantly shorter in the robot-assisted laparoscopy group compared with the conventional laparoscopy group (15 days vs. 13.0 days, respectively; *p* = 0.026). Conversion to open surgery was not necessary in either group. The International Prostate Symptom Score was significantly lower in the robot-assisted laparoscopy group compared with the conventional laparoscopy group. Moderate-to-severe symptoms were more frequently observed in the conventional laparoscopy group compared with the robot-assisted laparoscopy group (*p* = 0.051). Robotic surgery is safe and could improve functional disorder after rectal cancer surgery in the introduction phase. This may depend on the surgeon’s experience in performing robotic surgery and strictly confined criteria in Japan.

## Introduction

Rectal cancer is one of the most common malignant diseases worldwide [1]. Improvements in the prognosis of rectal cancer have been achieved by adjuvant and neoadjuvant chemotherapy; however, surgery is the mainstay of treatment and is the only method to cure rectal cancer.

Laparoscopic surgery for colon cancer is being increasingly used as a standard treatment as it has similar oncological outcomes to open surgery with potentially lower morbidity rates and better operative short-term and oncological outcomes [2, 3]. However, the superiority of laparoscopic surgery for rectal cancer is controversial.

Two large multi-center randomized clinical trials were unable to confirm the non-inferiority of laparoscopic surgery compared with open surgery in terms of the pathological completeness of resected specimens [4, 5]. Nevertheless, two major alternate trials report evidence that support the use of laparoscopic surgery in terms of pathological outcomes [6, 7]. It should be noted that these trials may not support laparoscopic resection for rectal cancer as a standard method of care.

Robotic assistance, which has recently been introduced in the field of surgery, is expected to overcome some of the limitations of laparoscopic surgery for rectal cancer. Robotic assistance uses articulating instruments that offer seven degrees of freedom of movement, immersive three-dimensional field depth, a stable camera platform, more precise surgical manipulation through tremor filtering and motion scaling, and greater ergonomic comfort in the operative environment [8, 9]. These features are useful for identification and preservation of small anatomical structures, such as the pelvic plexus, and precise total mesorectal excision (TME) in the narrow pelvic space. However, unexpected problems (robotic device and instrument malfunctions, patient injuries, and mortality) have been reported when introducing robotic surgery [10, 11].

A few non-randomized studies have suggested that robotic surgery may improve the quality of life of patients by preserving urinary and sexual function [12–14]. Some reports suggest that the learning curve in robotic surgery for rectal cancer is shorter when compared with laparoscopic surgery [15]. It is generally agreed that 15–35 cases are required to complete the learning curve in robotic surgery [15, 16], while 40–80 cases are required in laparoscopic surgery [17, 18]. Previous reports suggest that clinicopathological outcomes do not differ significantly in the introduction phase [19–21]; this is because surgeons can use the advantageous features of robotic surgery at the time of introduction.

To clarify the advantages and disadvantages of robotic surgery for rectal cancer in the introduction phase, we retrospectively reviewed cases of robotic surgery and conventional laparoscopic surgery.

## Methods

We first performed robotic surgery for rectal cancer in October 2015. We use the hybrid technique (robot-assisted TME), and only the anastomotic procedure is performed laparoscopically. We call “fusion surgery method”. Laparoscopic and robotic surgery were performed during the same period, thereby excluding the influence of time factor on the results. In Japan, robotic surgery has been covered by public health insurance since April 2018. Twenty-nine cases of robotic surgery were performed before public health insurance coverage was introduced. Before health insurance coverage was introduced, patients had to pay 500,000 yen, and patients themselves selected the method of surgery.

From the 16th case, we performed lateral pelvic lymph node dissection (LPLND) using robotic surgery [22]. In February 2017, robotic surgery with LPLND was introduced, and rectal cancer surgery with LPLND was performed using the laparoscopic method. Our indication criteria for LPLND were when the inferior tumor margin was located distal to the peritoneal reflection and extended beyond the muscularis propria layer, in accordance with the Japanese Society for Cancer of the Colon and Rectum guidelines [23]. Chemoradiotherapy is not widely used in Japan. Reports from Japan show an overall survival improvement with LPLND [24], and recent reports have shown benefit with LPLND after chemoradiotherapy [25].

Between 2015 and 2018, a total of 131 patients underwent surgery for rectal cancer at Sendai Medical Center. Conventional laparoscopic surgery was performed in 95 patients, and robotic surgery was performed in 36 patients.

This study retrospectively compared initial robotic surgery with laparoscopic surgery for rectal cancer, evaluated the clinical and pathological characteristics of patients, and examined the short-term outcomes and urinary and sexual function using a propensity score analysis.

The study protocol received local ethical approval from Sendai Medical Center (institutional review board No. 27–8), and written informed consent was obtained from all patients. We registered UMIN as a clinical trial [reference No.UMIN000019857].

Patients underwent clinical examinations, total colonoscopy, abdominopelvic computed tomography, and pelvic magnetic resonance imaging for preoperative staging. Patients with locally advanced cancer not amenable to curative surgery (clinical T4b), or suspected difficulty in securing the circumferential resection margin, received preoperative chemoradiotherapy and chemotherapy. Treatment decisions were reached by multidisciplinary meetings.

Self-reported questionnaires of urinary and sexual function were assessed for the International Prostate Symptom Score (IPSS) and International Index of Erectile Function (IIEF) for patients who underwent rectal cancer surgery preoperatively and 3, 6, and 12 months postoperatively. The IPSS was used to evaluate urinary function on a scale of 0–35, and higher scores indicated more severe symptoms. Urinary function was graded in three subgroups: normal function (IPSS, 0–7), moderate dysfunction (IPSS, 8–19), and severe dysfunction (IPSS, 20–35) [26]. In this study, an IPSS of ≥ 8 points was classified as moderate-to-severe dysfunction with lower urinary tract symptoms. Quality of life in the IPSS questionnaire was scored on a scale of 0 (best) to 6 (worst). Residual urine was measured after removal of the urethral catheter on postoperative day 5. A residual urine volume of ≥ 50 ml was regarded as urinary retention. Patients performed self-catheterization until the residual urine volume was < 50 ml [9]. The IIEF is a standardized male sexual function assessment with scores ranging from 5 to 75, and lower scores indicate more severe dysfunction [27].

### Surgery

Standardized principles and surgical procedures were used with both approaches. The only difference was that laparoscopic surgery was performed with left colic artery preservation [28], and robotic surgery was performed with high ligation of the inferior mesenteric artery (IMA) [29]. We introduced robotic surgery by initially inviting the robotic doctor in accordance with guidelines for introduction of robotic surgery from the Japan Society for Endoscopic Surgery. Therefore, we have done robotic surgery as the same method by Shizuoka Cancer Center procedure. Studies have reported no differences in the short-term results between left colic artery preservation and IMA ligation in anterior resection [30], so we decided to perform IMA ligation with robotic surgery.

All robotic surgeries were performed by a single operating surgeon (H.O.) using the da Vinci Si Surgical System® (Intuitive Surgical, Sunnyvale, CA, USA). We began to use robotic surgery in 2015 after performing more than 100 laparoscopic rectal resection procedures. Our robotic surgeon has experience in various laparoscopic surgeries, including hepatic resection, pancreatic resection, gastrectomy, and inguinal hernia surgery. The surgeon began to perform robotic surgery after participating in workshops and performing extensive experiments to obtain an animal laboratory and robotics certificate. The surgeon has 18 years of experience and a further 7 years of experience after obtaining the Endoscopic Surgical Skill Qualification System of the Japan Society for Endoscopic Surgery.

In our robotic procedure, we employed the so-called “dual docking method.” The patient cart was docked from the left caudal side at an angle of 30°–40°, and medial-to-lateral dissection with ligation of the inferior mesenteric artery and vein and mobilization of the descending and sigmoid colon were performed. Next, the patient cart was docked from between the patient’s legs, the robotic arms were docked again, and rectal mobilization down to the pelvic floor, TME, and LPLND were performed if necessary [22, 29]. Finally, the cart was moved away, and we performed conventional laparoscopic anastomosis. In cases of anterior resection (AR), we divided the distal rectum using linear staplers. We usually used an ECHELON FLEX™ GST System with a 60-mm gold or black cartridge (Ethicon Endo-Surgery, LLC., Cincinnati, OH, USA) or the Signia™ Stapling System or Tri-Staple™ 2.0 with reinforced reload (60-mm medium thickness) (Medtronic plc., Dublin, Ireland). The specimen was extracted through a 3–6-cm incision in the umbilical port. The anvil of the circular stapler was secured. We performed end-to-end anastomosis using the standard double-stapling technique (DST). We used the Proximate™ Intraluminal Stapler (CDH25A; Ethicon Endo-Surgery, LLC.) or the EEA™ Circular Stapler with DST series technology 25 × 4.8-mm staples. For inter-sphincteric resection (ISR), we performed transanal inter-sphincteric dissection and colo-anal hand sewn anastomosis. For abdominoperineal resection (APR), we performed perineal dissection of the pelvic diaphragm and sigmoid colostomy construction.

### Statistics

When comparing short-term outcomes, there are inherent biases in baseline clinical and pathological characteristics between patients undergoing laparoscopic and robotic surgery; thus, we used a 1:1 propensity score matching analysis. This method eliminates customary biases associated with conventional multivariate modeling approaches [31]. The covariate factors included the treating surgeon, age, body mass index (calculated as weight in kilograms divided by height in meters squared), tumor location, tumor size, pathological T category and lymph node metastasis, distant metastasis, use of preoperative chemoradiotherapy, surgical procedures, and LPLND. A paired* t* test was used to compare continuous data, and the Chi-squared test and Fisher’s exact test were used to compare categorical data. Statistical analyses were performed using JMP (SAS Institute Inc., Cary, NC, USA) and R (http://www.R-project.org/) software programs.* p* values of < 0.05 were considered statistically significant. Score changes in functional outcomes over 12 months were analyzed by repeated-measures analysis of variance. Significance was set at a two-sided* p* value of < 0.05.

## Results

The background features of patients treated with conventional laparoscopy and robot-assisted laparoscopy are shown in Table 1. With robot-assisted laparoscopy, the proportion of patients with upper rectal cancer was higher compared with the proportion of patients who underwent conventional laparoscopy.

**Table 1:** Clinical and surgical characteristics (laparoscopic vs. robotic surgery)

	Full cohort			Propensity score-m		
	Laparoscopic	Robotic	*p*	Laparoscopic	Robotic	*p*
*N*	95	36	0.019	36	36	
Age^a^	67.1	62.1	0.938	62.8	62.1	0.753
Male	60 (63.2%)	23 (63.9%)	0.142	23 (63.9%)	23 (63.9%)	1.000
BMI^a^ (kg/m^2^)	23.8	22.5	0.234	23.1	22.5	0.460
Location	26 (27.4%)	10 (27.8%)	0.421	10 (27.8%)	10 (27.8%)	1.000
Rs	29 (30.5%)	16 (44.4%)	0.165	16 (44.4%)	16 (44.4%)	0.495
Ra	40 (42.1%)	10 (27.8%)	0.529	10 (27.8%)	10 (27.8%)	0.806
Rb	40.6	44.0	0.779	40.6	44.0	0.951
Size (mm)	4 (4.2%)	1 (2.8%)	0.043	2 (5.6%)	1 (2.8.%)	0.642
T	22 (23.2%)	5 (13.9%)	0.501	8 (22.2%)	5 (13.9%)	0.745
is	12 (12.6%)	5 (13.9%)	0.331	3 (8.3%)	5 (13.9%)	0.151
1	46 (48.4%)	24 (66.6%)	0.025	22 (61.1%)	24 (66.6%)	0.457
2	11 (11.6%)	1 (2.8%)	0.250	1 (2.8%)	1 (2.8%)	1.000
3	57 (60.0%)	22 (61.1%)	0.280	24 (66.7%)	22 (61.1%)	0.743
4	26 (27.3%)	7 (19.4%)	0.155	6 (16.6%)	7 (19.4%)	0.586
*N*	11 (11.6%)	7 (19.4%)		6 (16.6%)	7 (19.4%)	0.586
0	1 (1.1%)	0(0%)		0 (0%)	0 (0%)	0.753
1	9 (9.5%)	3 (8.3%)		2 (5.6%)	3 (8.3%)	1.000
2	15 (15.8%)	13 (36.1%)		10 (27.7%)	13 (36.1%)	0.460
3	51 (53.7%)	18 (50.0%)		20 (55.6%)	18 (50.0%)	1.000
M (+)	27 (28.4%)	5 (13.9%)		6 (16.7%)	5 (13.9%)	0.495
ASA	2 (2.1%)	0 (0%)		0 (0%)	0 (0%)	0.806
1	3 (3.1%)	2 (5.6%)		0 (0%)	2 (5.6%)	0.951
2	30 (31.6%)	14 (38.9%)		11 (30.5%)	14 (38.9%)	0.642
3	26 (27.4%)	10 (27.8%)		10 (27.8%)	10 (27.8%)	0.745
4	47 (49.5%)	26 (72.2%)		26 (72.2%)	26 (72.2%)	0.151
Neoadjuvant chemotherapy	4 (4.2%)	0 (0%)		0 (0%)	0 (0%)	0.457
Adjuvant chemotherapy	6 (6.3%)	0 (0%)		0 (0%)	0 (0%)	1.000
Procedure	12 (12.6%)	0 (0%)		0 (0%)	0 (0%)	0.743
HAR	9 (9.5%)	6 (16.7%)		5 (13.8%)	6 (16.7%)	0.586
LAR	21 (22.1%)	10 (28.5%)		8 (22.2%)	10 (28.5%)	0.586
ISR	39 (41.5%)	10 (28.5%)		8 (22.2%)	10 (28.5%)	
Hartmann	95	36			36	
Mile’s	67.1	62.1			62.1	
LPLND (+)	60 (63.2%)	23 (63.9%)		23 (63.9%)	23 (63.9%)	
Covering stoma	23.8	22.5			22.5	
Stoma	26 (27.4%)	10 (27.8%)			10 (27.8%)	

Regarding the surgical procedures, only high anterior resection and low anterior resection were performed in initial cases of robot-assisted laparoscopy. After propensity score matching, the treatment groups were well balanced with respect to baseline characteristics and operative procedures (Table 1, propensity score-matched cohort).

We compared the short-term outcomes among the propensity score-matched treatment groups (Table 2). The mean surgical duration was significantly longer in the robot-assisted laparoscopy group than the conventional laparoscopy group (288.4 vs. 245.2 min, respectively; *p* = 0.051). In patients who underwent LPLND, the surgical duration was comparable between groups (508.0 min for robot-assisted laparoscopy vs. 480.4 min for conventional laparoscopy; *p* = 0.595). Compared with the conventional laparoscopy group, the estimated volume of blood loss was lower in the robot-assisted laparoscopy group (38.2 ml vs. 16.3 ml, respectively; *p* = 0.169).

None of the patients required conversion in either group. No significant difference was found between the groups in overall morbidity rate (Table 2). A comparable outcome for surgical oncology, including the positive radial margin (RM) rate, was obtained between groups. The length of hospital stay was significantly longer in the conventional laparoscopy group compared with the robot-assisted laparoscopy group (15.4 days vs. 13.0 days, respectively; *p* = 0.026).

**Table 2:** Short-term outcomes (laparoscopic vs. robotic surgery)

	Full cohort			Propensity sc		
	Laparoscopic	Robotic	*p*	Laparoscopic	Robotic	*p*
*n*	95	36	0.204	36	36	0.095
Conversion	0	0	0.142	0	0	0.0506
Operation time^a^ (min)	295.8 (140–618)	326.0 (172–595)	0.142	278 (144–618)	326 (172–595)	0.595
−LPLND	277.3 (140–591)	288.4 (172–572)	0.086	245.2 (144–395)	288.4 (172–572)	0169
+LPLND	472.7 (390–618)	508.0 (469–595)	0.0017	480.4 (329–618)	508 (469–595)	**0.026**
Blood loss^a^ (ml)	57.8 (1–895)	16.3 (1–103)	0.870	38.2 (1–643)	16.3 (1–103)	0.641
Hospital stay^b^ (day)	17.3	13.0	0.971	15.4	13.0	0.286
RM(+)	6 (6.3%)	2 (5.6%)	0.811	3 (8.3%)	2 (5.6%)	1.000
Distal margin^b^ (mm)	37.5	37.8	0.543	30.0	37.4	0.555
Complication	23 (24.2%)	8 (22.2%)	0.910	8 (22.8%)	8 (22.2%)	0.313
SSI	5( 5.2%)	1 (2.8%)	0.543	2 (5.7%)	1 (2.8%)	0.303
Anastomotic leak	3 (3.2%)	1 (2.8%)	0.327	0 (0%)	1 (2.8%)	0.303
Ileus	5 (5.2%)	1 (2.8%)	–	3 (8.5%)	1 (2.8%)	–
Urinary retention (50ml>)	7 (7.4%)	1 (2.8%)		3 (8.5%)	1 (2.8%)	0.095
Others	3 (3.2%)	4 (11.1%)		0 (0%)	4 (11.1%)	0.0506

Among 131 patients, 14 patients (38.9%) in the robot-assisted laparoscopy group and 30 patients (31.6%) in the conventional laparoscopy group received adjuvant chemotherapy. Two patients (5.6%) in the robot-assisted laparoscopy group and three patients (3.2%) in the conventional laparoscopy group received neoadjuvant chemotherapy, with no difference between the two treatment groups.

Urinary function.

**Fig. 1:**
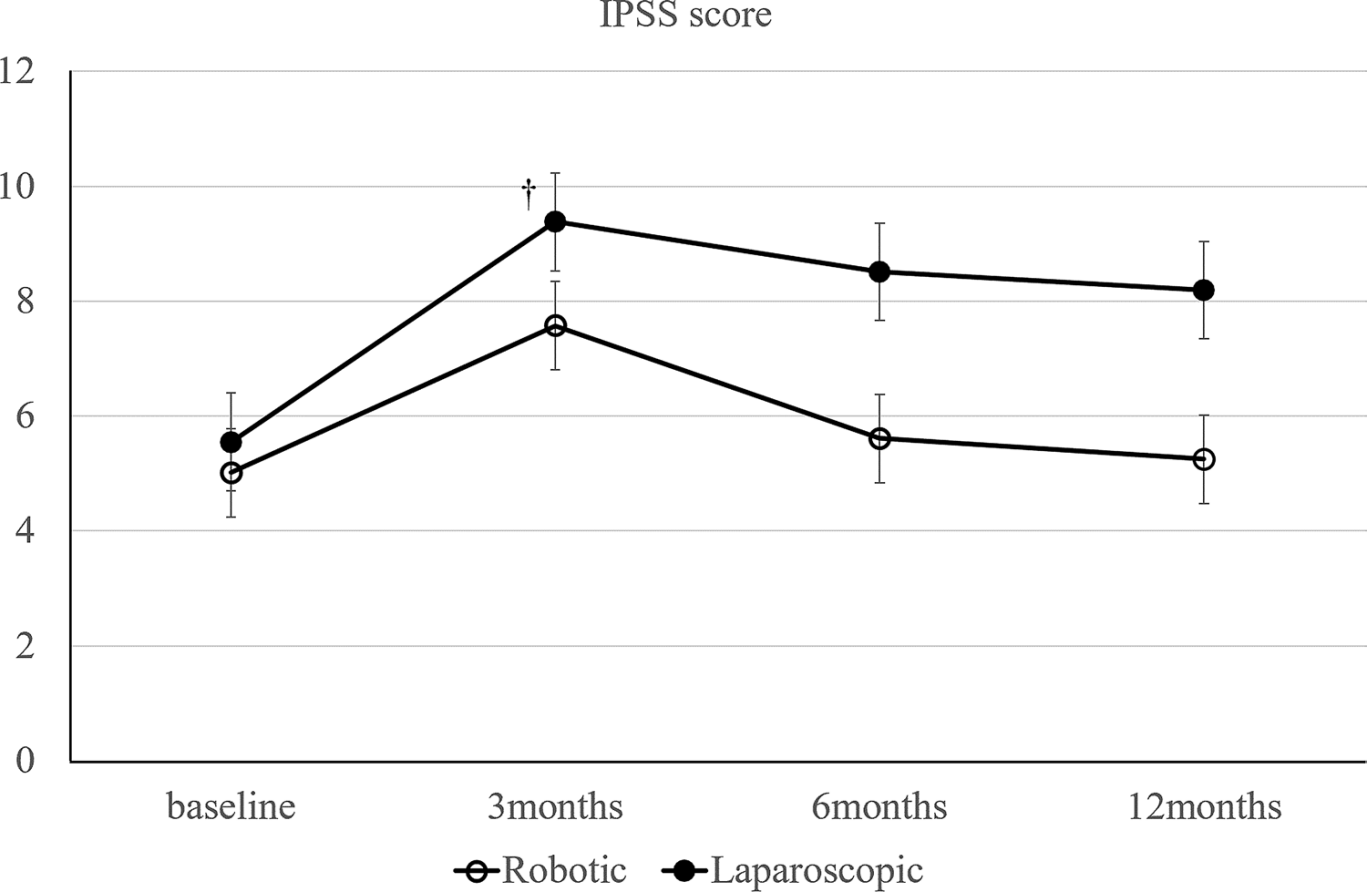
Change in urinary function (IPSS). *p < 0.05 for differences in mean scores between groups. †*p* < 0.05 for differences in mean scores between baseline and each point

Urinary function was similar in both groups according to baseline IPSS. The scores increased 3 months after surgery, and gradually decreased thereafter. The IPSS recovered to nearly the same level as baseline in the robot-assisted laparoscopy group at 12 months, but not in the conventional laparoscopy group (Fig. 1).

The IPSS was significantly lower in the robot-assisted laparoscopy group compared with the conventional laparoscopy group 12 months after surgery in the entire cohort (Table 3). However, these differences disappeared after propensity score matching (Table 3). In particular, the proportion of patients with an IPSS of ≥ 8 points (classified as having moderate-to-severe symptoms) tended to be higher in the conventional laparoscopy group (Table 3).

**Table 3:** Comparison of IPSS score between laparoscopic and robotic surgery 12 months after the operation

	Full cohort			Propensity sc		
	Laparoscopic	Robotic	*p*	Laparoscopic	Robotic	*p*
*n*	59	20	0.049	26	20	0.119
IPSS^a^	8.81	5.25	0.008	8.19	5.25	0.051
IPSS>8	17 (28.81%)	2 (10%)	0.476	7 (26.9%)	2 (10%)	0.778
IPSS QOLscore^a^	2.38	2.16		2.14	2.16	

Sexual function in male patients.

After propensity score matching, 22 sexually active males were included from each group; sexual function was not analyzed in women because of low response rates.

**Fig. 2:**
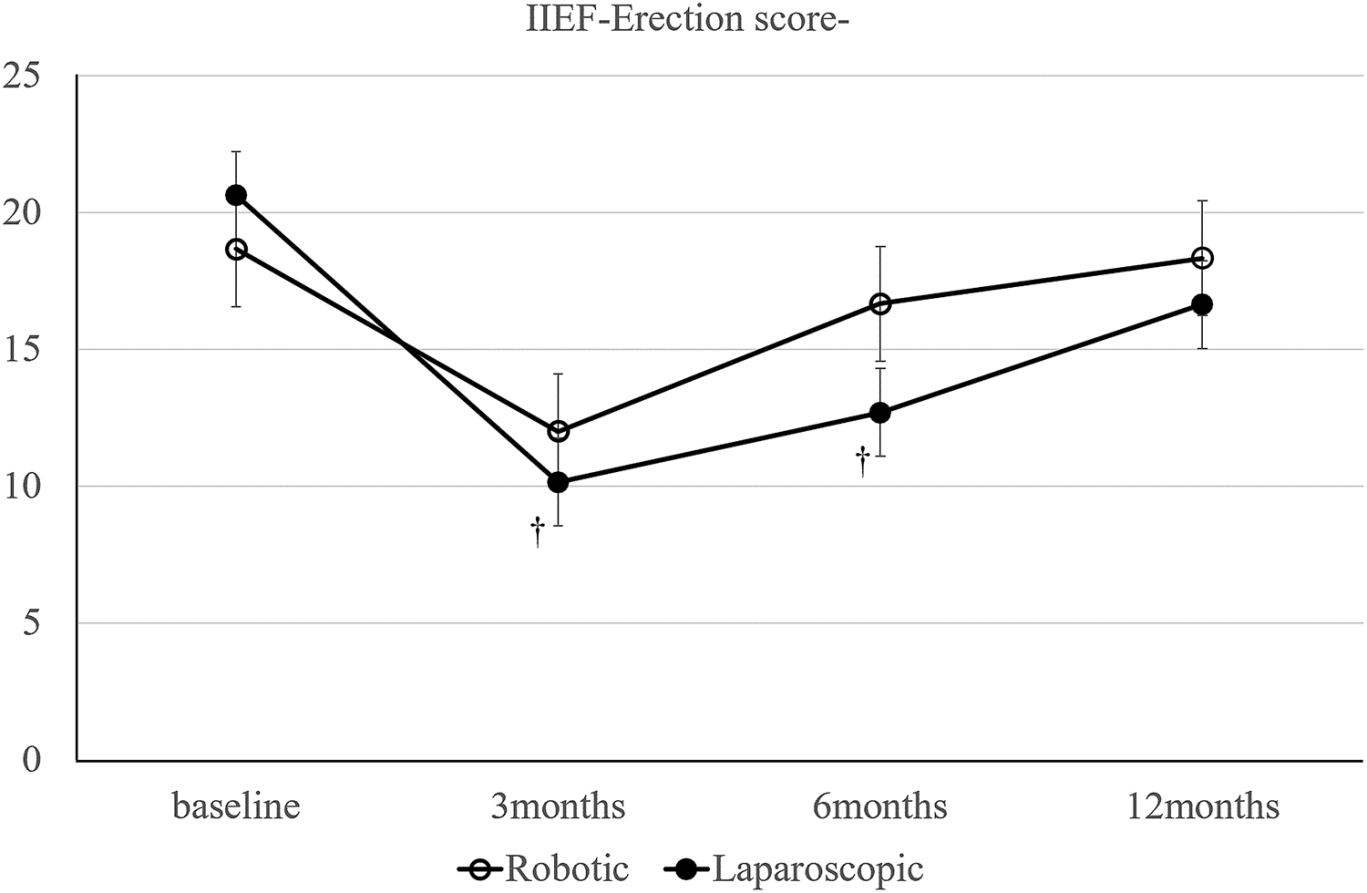
Change in erectile function (IIEF erection score). *p < 0.05 for differences in mean scores between groups. †*p* < 0.05 for differences in mean scores between baseline and each point

**Fig. 3:**
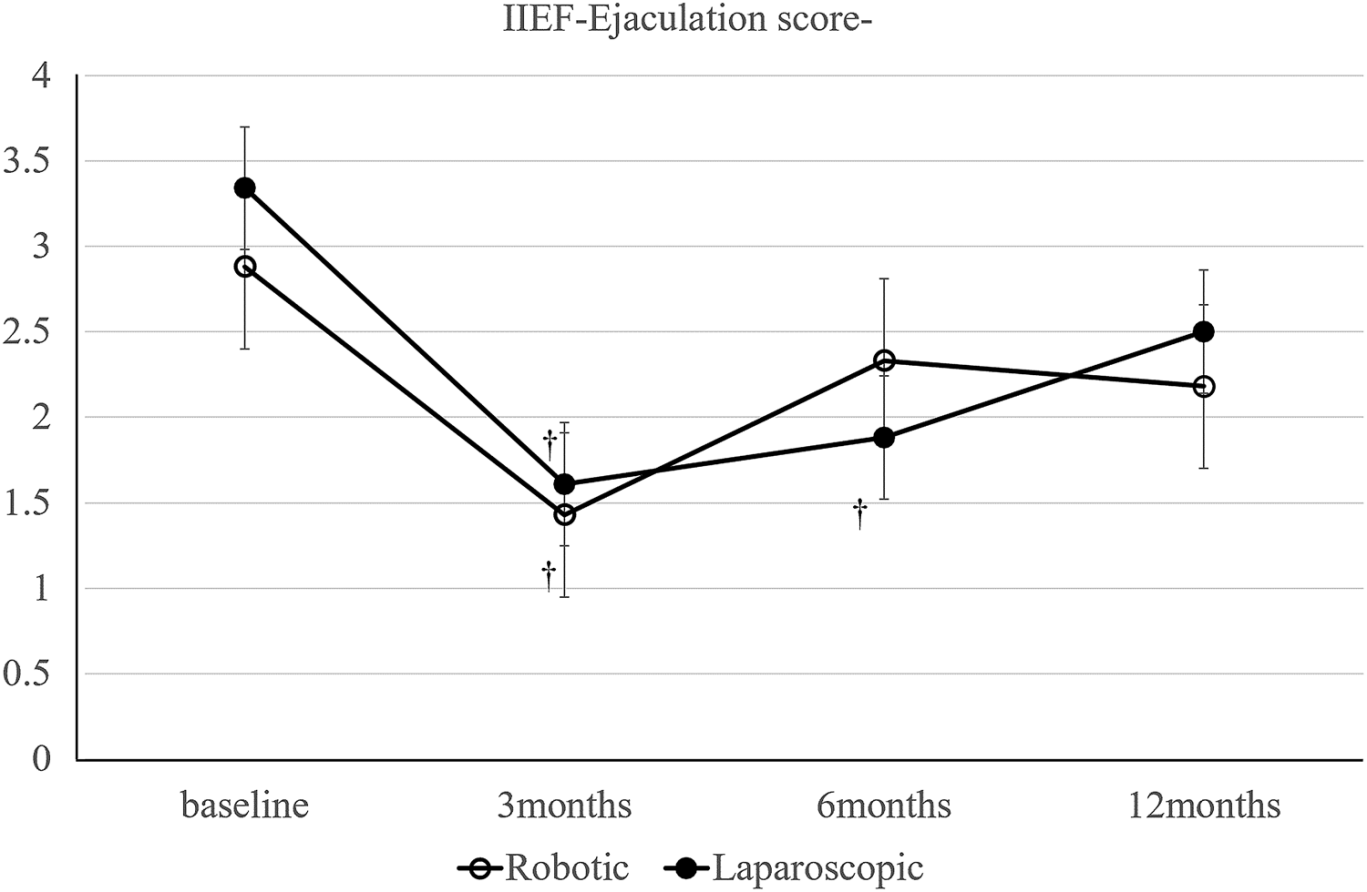
Change in ejaculatory function (IIEF ejaculation score). *p < 0.05 for differences in mean scores between groups. †* p* < 0.05 for differences in mean scores between baseline and each point

The IIEF score was not significantly different between groups throughout the study period. We examined the IIEF erection and ejaculation scores separately but neither showed a statistically significant difference between groups (Figs. 2, 3).

## Discussion

Some reports have assessed short-term surgical outcomes [9, 29, 32] during the trial stage of robotic surgery; however, there are few reports on functional disorders in the trial stage of robotic surgery. We examined dysfunction in initial cases of robotic surgery and postoperative short-term outcomes to compare laparoscopic surgery in the same period using propensity score matching. The findings of this comparative study show that robotic surgery decreased the length of postoperative hospitalization, the rate of urinary retention, the IPSS 12 months after surgery, and the proportion of IPSS classified as moderate to severe compared with conventional laparoscopy. These data suggest that robot-assisted laparoscopy for rectal cancer might improve functional disorder in the introductory phase.

The long duration of robot-assisted laparoscopy could be explained by the time taken to set up and dock the robotic system and arrange the robotic arms. It is important to prevent collisions by proper positioning of robotic ports and proper manipulation of robotic forceps. Considering that the duration of robot-assisted laparoscopy included the setup time, the time taken for surgical manipulation may be shorter with robot-assisted laparoscopy vs. conventional laparoscopy. The length of time using forceps may also be shorter with robot-assisted laparoscopy. In patients who underwent LPLND, the surgical duration was similar between robot-assisted laparoscopy and conventional laparoscopy. This result suggests that robot-assisted laparoscopy may be effective in narrow areas close to larger vessels and nerves. The duration of robot-assisted laparoscopy will inevitably decrease with the accumulation of experience [15]. As of April 2020, we have performed more than 100 robot-assisted laparoscopy procedures for rectal cancer, and the surgical duration was equivalent to that of conventional laparoscopy (data not shown).

In the entire study period, we did not experience conversion to open surgery. The ROLARR trial suggested a trend towards fewer cases of conversion with robotic surgery compared with conventional laparoscopy [33]. The robotic system allows surgeons to perform safer dissection in a narrow pelvic cavity. Additionally, the ROLARR trial reported that the conversion rate of robotic-assisted surgery was lower when performed by experienced surgeons who had performed more than 180 robotic-assisted laparoscopies compared with surgeons who had performed less than 45 robotic-assisted laparoscopies, regardless of their level of conventional laparoscopic experience. The main reasons for conversion to laparotomy were difficulty in exposing the operative field, uncontrollable bleeding, and damage to other organs. Even in initial cases (< 45 cases), robotic surgery can provide a number of advantages, even in the introductory phase. In Japan, robotic surgeons are selected using strict operator criteria (http://www.jses.or.jp/member/robot_assisted_surgery.html) such as attaining the Endoscopic Surgical Skill Qualification System of the Japan Society for Endoscopic Surgery, employing a board-certified surgeon in gastroenterology, and obtaining a certificate of robotic surgery. We also need to have experience in more than 20 cases of laparoscopic rectal cancer surgery.

Surgeons must be assessed according to the Endoscopic Surgical Skill Qualification System of the Japan Society for Endoscopic Surgery [34, 35]. Surgeons are selected according to very strict criteria when introducing robotic surgery. Therefore, it is guaranteed that a very adept specialist will perform the procedure, leading to a conversion rate of 0% from the trial stage. Conversion is associated with postoperative complications and morbidity [2]. Furthermore, conversion reduces the survival rate in laparoscopic surgery for colorectal cancer [36]. This finding is also observed in rectal cancer [2, 36]. The low conversion rate is crucial for oncological outcomes of both robotic-assisted and conventional laparoscopic surgery. We are convinced that our strict operator criteria will lead to exemplary results (http://www.jses.or.jp/member/robot_assisted_surgery.html).

The rate of complications was similar between groups (22.8% for robot-assisted laparoscopy vs. 22.8% for conventional laparoscopy, *p* = 1.00). The length of postoperative hospital stay was significantly longer in the conventional laparoscopy group compared with the robot-assisted laparoscopy group (15 days vs. 13.0 days, respectively; *p* = 0.026). This may be attributed to low postoperative urinary retention (8.5% for conventional laparoscopy vs. 2.8% for robot-assisted laparoscopy; *p* = 0.303) and ileus (8.5% for conventional laparoscopy vs. 2.8% for robot-assisted laparoscopy; *p* = 0.303) rates with robot-assisted laparoscopy. In this study, the rate of anastomotic leakage when performed by qualified surgeons was as low as 2.4%. In contrast, when a qualified surgeon was not involved in the surgery, the rate of anastomotic leakage increased to 20%. Our results support the benefits of the operator’s criteria for robotic surgery in Japan.

In Japan, the adequacy of resection margins is evaluated using the RM rate, as defined in the Japanese Classification of Colorectal, Appendiceal, and Anal Carcinoma [37]. We showed a similar RM rate in both groups, which was higher compared with the results of other Japanese institutions [15, 32] and Korean institutions [6]. This might be because this study included T4 cases (*n* = 12), which are associated with a high risk of RM involvement. After excluding T4 cases, the RM positive rate was 2.8% in the robot-assisted laparoscopy group and 3.1% in the conventional laparoscopy group, which is equivalent to the results reported by other studies.

LPLND is technically difficult in areas close to large vessels and nerves in the narrow complex area of the pelvis. There are also reports of LPLND performed laparoscopically [38]. However, it is a difficult procedure, because LPLND is performed using rigid forceps and straight forceps without joints; thus, operability is not sufficient. We think that the unparalleled advantage of robotic surgery with LPLND is the ability to perform optimum and precise traction of the lymph nodes; this manipulation reveals the dissectible layer to be removed. Robotic surgery is expected to improve the operability of LPLND through its numerous benefits [22].

Previous studies suggest that recovery of bladder and sexual function occurs earlier with robot-assisted laparoscopy compared with conventional laparoscopy [12, 14, 39, 40]. These functions usually improve 12 months after surgery, and symptoms are fixed during this period. Our results were similar to previous studies (Figs. 1, 2, 3). Particularly with the IPSS, the proportion of patients who scored ≥ 8 points and who were, therefore, classified as having moderate-to-severe symptoms tended to be higher in the conventional laparoscopy group. This result suggests that robot-assisted laparoscopy may improve urinary retention and other functions related to urinary flow, bladder compliance, and detrusor activity. Urinary dysfunction is related to damage of the hypogastric, parasympathetic, sympathetic, and pudendal nerves during surgery. Nerve injury causes crosstalk and urinary dysfunction.

Kanao reported that urinary dysfunction changes depending on the location of pelvic plexus injury [41]. Kim reported that the cause of urinary dysfunction might be temporary nerve injury due to traction or diathermy injury or incomplete division of nerves that will later spontaneously regenerate [14]. Furthermore, other studies suggest that identification of pelvic autonomic nerves in rectal cancer surgery is very important; this is because if nerves are not observed during surgery, this could lead to severe urinary dysfunction, which is responsible for the requirement for continued catheterization in 8.8%–38.5% of patients [42, 43]. Furthermore, nerve dissection during surgery causes catheterization in 30.8%–53% of patients [44, 45]. These previous studies report that severe nerve injury or dissection causes dysfunction for up to 12 months postoperatively. However, robot-assisted laparoscopy uses a stable three-dimensional magnified view that can be controlled by itself, which improves visualization of the narrow deep pelvic field. Additionally, the second and third robotic arms facilitate stable and adequate countertraction and are useful for exposing the surgical field [8]. This may be useful for preventing nerve damage due to hyperextension or diathermy injury. Furthermore, sexual dysfunction suggests damage, especially in the neurovascular bundle and cavernous nerve [46]. These anterolateral areas are susceptible to injury in the narrow pelvic area when using rigid instruments in laparoscopic surgery for the fulcrum effect. Robotic surgery facilitates precise control of these neurovascular bundles for anterolateral dissection [14].

This study has several limitations. First, this study included a small number of cases and adopted a retrospective non-randomized design. However, we conducted a propensity score matching analysis to reduce bias between groups. Second, all robotic surgeries were performed by a single surgeon. Thus, the results may not be generalizable to all situations. Third, we did not analyze defecation or female sexual function in this study. Because we used a small-sample size, we are now collecting data on these functions. Finally, long-term oncological results were not evaluated in this study. The incidence of local recurrence, progression-free survival, and overall survival should also be evaluated in the future to assess the true advantages of robotic surgery for rectal cancer.

## Conclusion

Robotic surgery was performed safely and may improve functional disorder in the introductory phase. This result may depend on the surgeon performing robotic surgery and on strictly confined criteria in Japan.

## Data Availability

Data can be made available on request.
